# Octopamine is required for successful reproduction in the classical insect model, *Rhodnius prolixus*

**DOI:** 10.1371/journal.pone.0306611

**Published:** 2024-07-12

**Authors:** Jimena Leyria, Ian Orchard, Angela B. Lange

**Affiliations:** Department of Biology, University of Toronto Mississauga, Mississauga, ON, Canada; University of Leipzig Faculty of Life Sciences: Universitat Leipzig Fakultat fur Lebenswissenschaften, GERMANY

## Abstract

In insects, biogenic amines function as neurotransmitters, neuromodulators, and neurohormones, influencing various behaviors, including those related to reproduction such as response to sex pheromones, oogenesis, oviposition, courtship, and mating. Octopamine (OA), an analog of the vertebrate norepinephrine, is synthesized from the biogenic amine tyramine by the enzyme tyramine β-hydroxylase (TβH). Here, we investigate the mechanisms and target genes underlying the role of OA in successful reproduction in females of *Rhodnius prolixus*, a vector of Chagas disease, by downregulating *TβH* mRNA expression (thereby reducing OA content) using RNA interference (RNAi), and in vivo and ex vivo application of OA. Injection of females with dsTβH impairs successful reproduction at least in part, by decreasing the transcript expression of enzymes involved in juvenile hormone biosynthesis, the primary hormone for oogenesis in *R*. *prolixus*, thereby interfering with oogenesis, ovulation and oviposition. This study offers valuable insights into the involvement of OA for successful reproduction in *R*. *prolixus* females. Understanding the reproductive biology of *R*. *prolixus* is crucial in a medical context for controlling the spread of the disease.

## Introduction

In insects, biogenic amines, including octopamine (OA), tyramine (TA), dopamine (DA), and serotonin (5-HT), can act as neurotransmitters, enabling transmission across the synaptic cleft, as neuromodulators, influencing nearby cells, and/or as neurohormones, being transported through the hemolymph and exerting effects over greater distances and multiple tissues. They can mediate rapid and/or longer-term behavioral changes in response to shifting environmental and physiological conditions [1–4]. Biogenic amines are expressed in a variety of cell types such as central and peripheral neurons, neurosecretory and sensory neurons, and endocrine cells in the gut [3]. Typically, their mode of action involves G protein-coupled receptors (GPCRs) that mediate changes in the intracellular levels of the second messenger cyclic AMP and/or with the generation of intracellular calcium signals. The location of these GPCRs serves to identify the target sites of the respective amine [5–7]. Biogenic amines modulate various insect behaviors, including those involved in reproduction, such as responses to sex pheromones, oogenesis, oviposition, courtship, and mating [7–9].

OA, an analog of norepinephrine first identified in the salivary gland of the octopus *Octopus vulgaris* [10], is produced from the amino acid tyrosine. Tyrosine decarboxylase (TDC) facilitates the conversion of tyrosine to TA by decarboxylation. Subsequently, tyramine β-hydroxylase (TβH) converts TA to OA through hydroxylation [11, 12]. OA receptors are classified as two α-adrenergic-like OA receptors (OAα1R, OAα2R) and three β-adrenergic-like OA receptors (OAβ1R, OAβ2R, OAβ3R) [13]. Alternative splicing of the OAα2R transcript has been identified in some species [6, 14]. Interestingly, TA can also bind to OA receptors with varying affinities [13]. One of the main functions of OA in insects is as a stress hormone, i.e. flight or fight hormone, regulating the insect’s energy demands as needed [1]. OA is also involved in other behaviors such as modulating locust flight, aggression and wakefulness in flies, and learning and memory [1, 11]. It also plays important roles in reproductive physiology modulating aspects of ovulation and oviposition [7, 15–20], and also oogenesis, by influencing the titer of the lipophilic sesquiterpenoid juvenile hormone (JH) or the main yolk protein precursor, vitellogenin (Vg) [21–27]. JH is primarily produced by, and secreted from, the corpus allatum (CA), a small gland located in the retrocerebral complex associated with the insect brain, and in close proximity with another neuroendocrine organ, the corpus cardiacum (CC) [28–30]. In insects, JH production represents the culmination of intricate crosstalk among various neurohormones (neuropeptides and biogenic amines) and hormones [29, 31–35].

*Rhodnius prolixus*, the blood gorging kissing bug, is a model insect extensively used for neuroendocrinological studies [19, 36, 37]. It is also medically important, being a principal vector of *Trypanosoma cruzi*, the causative agent of Chagas disease in humans [38, 39]. In the adult *R*. *prolixus* female reproductive system there are two ovaries, each containing 7 ovarioles where oogenesis (egg development) occurs [19, 40]. Oogenesis, which typically begins after a blood meal, is primarily regulated by JH through a receptor complex consisting of Methoprene tolerant (Met) and Taiman (Tai) proteins [19, 35, 41]. Once the egg is formed and chorionated (around 5–6 days after oogenesis begins), ovulation occurs. The egg moves from the ovaries into the lateral oviduct via the calyx, an expansion of the lateral oviduct into which the seven ovarioles open. The egg then travels along the common oviduct, passing the spermatheca, from which sperm are released onto the egg for fertilization, and then arrives at the bursa (genital chamber), ultimately leading to asynchronous oviposition for around 25–30 days. Females can feed several more times, initiating a new reproductive cycle with each feeding [19]. Although the five OA-like receptors have been identified in *R*. *prolixus* [19], only two of them have been confirmed to modulate successful reproduction [42–44]. OA facilitates the relaxation of the oviducts and the bursa, events associated with ovulation and oviposition, mainly via a β-adrenergic receptor, OAβ2R [42, 43], and modulates oogenesis and choriogenesis via an α-adrenergic receptor, OAα1R [44].

TβH transcriptional levels and enzyme activity have been reported to be closely correlated with OA titers. In the fly fruit *Drosophila melanogaster*, the red flour beetle *Tribolium castaneum*, the brown planthopper *Nilaparvata lugens*, and the oriental armyworm *Mythmina separata*, OA levels decrease when *TβH* expression is downregulated or mutated [27, 45–48]. Here, we elucidate the mechanisms and target genes underlying OA action, which results in successful reproduction in *R*. *prolixus* females, by downregulating the biosynthetic enzyme TβH using RNA interference (RNAi). When females are injected with double stranded TβH (dsTβH), egg production (eggs made and laid) is impaired, possibly due to a decrease in the expression of enzymes involved in JH biosynthesis, and ovulation and/or oviposition are interfered with, as shown by a retention of eggs in the calyx and less eggs laid. Ex vivo and in vivo application of OA supports the involvement of OA in these events. This work offers insights into the reproductive physiology of *R*. *prolixus* that may contribute to the development of novel approaches for insect pest management.

## Material and methods

### Experimental animals

*R*. *prolixus* obtained from a well-established colony at the University of Toronto Mississauga, were maintained at 25°C under elevated humidity levels (∼50%). Male and female insects were separated in their final (fifth) nymphal instar. At 30 days post-ecdysis (d PE), fifths were fed on defibrinated rabbit blood (Cedarlane Laboratories Inc., Burlington, ON, Canada) according to Orchard et al., [49]. Only insects that ingested at least 8 to 9 times their initial body weight (a typical meal size for fifth instars that initiates growth and development into the adult) were used in the experiments. Unless specified otherwise, for all experiments, adult females 1–2 d PE were individually isolated and placed with two recently fed males. Mating success was confirmed by inspecting the cubicle for deposited spermatophores [50]. When required, females 10 d PE were fed, and only those that consumed 2.5 to 3 times their initial body weight (typical of adult females to initiate a reproductive cycle) were selected for experiments. In all cases, blood consumption was measured by weighing the insect immediately before and after feeding [33, 41, 51].

### Phylogenetic analysis of tyramine β-hydroxylase

The evolutionary history was inferred by using the Maximum Likelihood method and Jones-Taylor-Thornton (JTT) matrix-based model [52]. The initial tree for the heuristic search was obtained automatically by applying Neighbor-Join and BioNJ algorithms to a matrix of pairwise distances estimated using the JTT model, and then selecting the topology with superior log likelihood value. The proportion of sites where at least 1 unambiguous base is present in at least 1 sequence for each descendent clade is shown next to each internal node in the tree. This analysis involved 18 amino acid sequences. There was a total of 659 positions in the final dataset. Evolutionary analyses were conducted in MEGA11 [53].

### RNA extraction and reverse transcription/quantitative PCR (RT-qPCR)

Total RNA extraction from fat body, ovaries, or CNS-CC-CA complexes was performed using TRIzol reagent (Invitrogen by Thermo Fisher Scientific, Waltham, MA, USA) following the manufacturer’s instructions. First-strand complementary DNA (cDNA) synthesis, using 1 μg total RNA, and qRT-PCR assays were carried out as previously detailed [33, 41]. Reference genes, including actin, Rp49 (60S ribosomal protein L32), or 18S ribosomal RNA (18S rRNA), were utilized for normalization of target gene expression (S1 Table). Primer pairs for each target gene exhibited an efficiency ranging from 85 to 110%, linear correlation coefficients (r^2^) from 0.8 to 1, and dissociation curves consistently showing a single peak. Tissue distribution and temporal mRNA expression were calculated relative to 1000 copies of the average of the reference genes using the 2^−ΔCt^ method [54]. For some experiments, results were presented as fold change relative to the expression of control samples, utilizing the 2^−ΔΔCt^ method following the geometric mean of the reference genes [55]. Experiments were repeated with at least 4–5 independent biological replicates, as indicated in each experiment, having two technical replicates and using no-template controls.

### OA treatment: In vivo and ex vivo experiments

For in vivo assays, adult virgin females (3 d PE) were injected through the dorsal intersegmental membrane with 5 μL of 10^−4^ M OA (D, L-octopamine hydrochloride from Millipore-Sigma, Oakville, Canada) in saline (150 mM NaCl, 8.6 mM KCl, 2 mM CaCl_2_, 4 mM NaHCO_3_, 34 mM glucose, 8.5 mM MgCl_2_, 5 mM HEPES [pH 7.2]), or 5 μL of saline for controls. Fat body and central nervous system (CNS) with the retrocerebral complex attached (CNS-CC-CA) were dissected 12 h later and processed for RT-qPCR. The results are shown as the mean ± SEM of n = 5–6 independent biological replicates. For the egg laying assay, fed mated females 6 days post-blood meal (PBM) were injected through the dorsal intersegmental membrane with 5 μL of 10^−4^ M OA in saline or 5 μL of saline for controls. Egg laying was recorded throughout 14 d PBM (n = 10–15 females). For ex vivo assays, CNS-CC-CA from adult virgin females (3 d PE) were incubated in 1.5 mL microtubes containing 200 μL of Grace’s medium, with L-glutamine (Millipore-Sigma, Oakville, ON, Canada), as previously described [33]. Two μL of 10^−3^ M OA, diluted in saline, was added to the medium (final concentration of 10^−5^ M); 2 μL of saline was added for controls. CNS-CC-CA were collected after 6 h of incubation and processed for RT-qPCR. The results are shown as the mean ± SEM of n = 4–5 independent biological replicates. In all cases, the final timepoints for the experiments were chosen based on the optimal changes in transcript expression observed after neurohormonal challenge and the viability of the tissues, as reported previously [33, 41, 56].

### RNAi assay

To knock down the *TβH* transcript, two non-overlapping fragments were generated. Double-stranded RNA (dsRNA) was synthesized using the T7 Ribomax Express RNAi System (Promega, WI, USA). Gene-specific primers are listed in S1 Table. All the controls were injected with a dsRNA encoding the partial sequence of the ampicillin resistance gene (dsARG), which targets a transcript absent in *R*. *prolixus*. Five μg of each dsRNA was injected into the hemocoel of females 3 d PE. Treated insects were given a blood meal at 10 d PE as detailed above. Insects were monitored for egg laying and subsequent hatchability. Tissues were also collected 6 d PBM for RT-qPCR, SDS-PAGE and western blot, and ovarian morphology analysis.

### Egg laying, egg volume and hatching ratio analysis

To analyze the ovarian morphology, ovaries were dissected and photographed with a digital microscope (Leica DVM6, Wetzlar, Germany). Egg laying after dsRNA treatment was observed for a period of 18 d PBM (n = 10 to 15 females). The total cumulative eggs laid per female were collected after 28 d PBM (n = 15 females) [41]. The volume of eggs (mm^3^) was determined using the formula for the volume of an ellipsoid considering the circular nature of the longitudinal axis: (1/6) π length width^2^ (n = 15) [44]. For hatching rate, eggs from control or dsTβH-injected females were collected, and the number of nymphs that hatched was recorded (n = 150 eggs).

### Protein measurements

Ovaries and fat bodies (ventral and dorsal) were dissected in cold *R*. *prolixus* saline and homogenized in 1.5 mL microtubes containing 200 μL of cold phosphate-buffered saline (20 mM Na_2_HPO_4_/KH_2_PO_4_, 150 mM NaCl, pH 6). The samples were then centrifuged at 2,500 × g for 5 min at 4°C; the resultant supernatant was centrifuged again at 12,000 × g for 10 min at 4°C. The supernatant from the second centrifugation was collected for protein quantification. The results are shown as the mean ± SEM of n = 5–6 independent biological replicates.

Ten μL of hemolymph (from 1 insect) was collected with a Hamilton syringe (Hamilton Company, Reno, NV, United States) from the cut ends of the legs while gently pressing on the abdomen. The hemolymph was transferred to ice-cold microtubes and then diluted in cold anticoagulant solution (composed of 10 mM Na_2_EDTA, 100 mM glucose, 62 mM NaCl, 30 mM sodium citrate, 26 mM citric acid, pH 4.6) at a ratio of 1:5 (anticoagulant: hemolymph) [57]. The samples were then centrifuged at 10,000 × g for 10 min at 4°C to remove debris, and the resulting supernatants were utilized for protein quantification. The results are shown as the mean ± SEM of n = 7–8 independent biological replicates.

Protein determination was performed using the BCA protein quantification assay (Pierce™ BCA Protein Assay Kit, Thermo Fisher Scientific, Mississauga, ON, Canada).

### SDS-PAGE and western blot

Tissues and hemolymph (10 μg/line) obtained as described previously, from dsRNA-treated insects were separated under reducing conditions on pre-made gels (4–20%, Mini-Protean TGX Stain-Free Precast Gels, BioRad, Mississauga, ON, Canada). The gels were stained with QC Colloidal Coomassie (BioRad) for 1 h at room temperature with gentle shaking and imaged on a ChemiDoc XRS system (BioRad). For western blot, tissues and hemolymph (5 μg/line) proteins from each dsRNA treatment were separated under reducing conditions on pre-made gels. A detailed protocol for western blot was described previously [51]. The polyclonal anti-Vg antibody was purchased from Boster Biological Technology (Pleasanton, CA, USA). Primary antibody (1:2000 dilution in PBS-T (PBS containing 0.1% Tween-20)) with 3% bovine serum albumin (BSA) was incubated overnight at 4°C with gentle shaking. HRP-conjugated goat anti-rabbit IgG (secondary antibody,1:5000 dilution in PBS-T with 3% BSA) was incubated for 1 h at room temperature. Blots were visualized using enhanced chemiluminescence (Clarity Western ECL Substrate, BioRad), imaged on a ChemiDoc XRS system, and analyzed using Image Lab 5.0 (BioRad Software and System). After stripping with RestoreTM PLUS Western blot Stripping buffer (Thermo Fisher Scientific, Mississauga, ON, Canada), the blots were re-probed with the anti-tubulin antibody (mouse monoclonal antibody from Life Technologies, ON, CA). The specificity of the primary antibody was already reported by Leyria et al., [41]. The standards (Precision Plus Protein™ Dual Xtra Prestained Protein Standards, BioRad) were detected by fluorescence when excited at red and green wavelengths.

### Statistical analyses

All data were analyzed using GraphPad Prism Software (GraphPad Software, San Diego, CA, USA). All datasets passed normality and homoscedasticity tests, ensuring that the assumptions required for performing a Student’s t-test were met. Significant differences were determined either using a one-tailed Student’s t-test or a one-way or repeated-measures two-way ANOVA followed by the post-hoc Tukey’s test or Bonferroni’s test, as indicated.

## Results

### *TβH* transcript is almost exclusively expressed in the CNS-CC-CA of *R*. *prolixus* females

**Fig 1:**
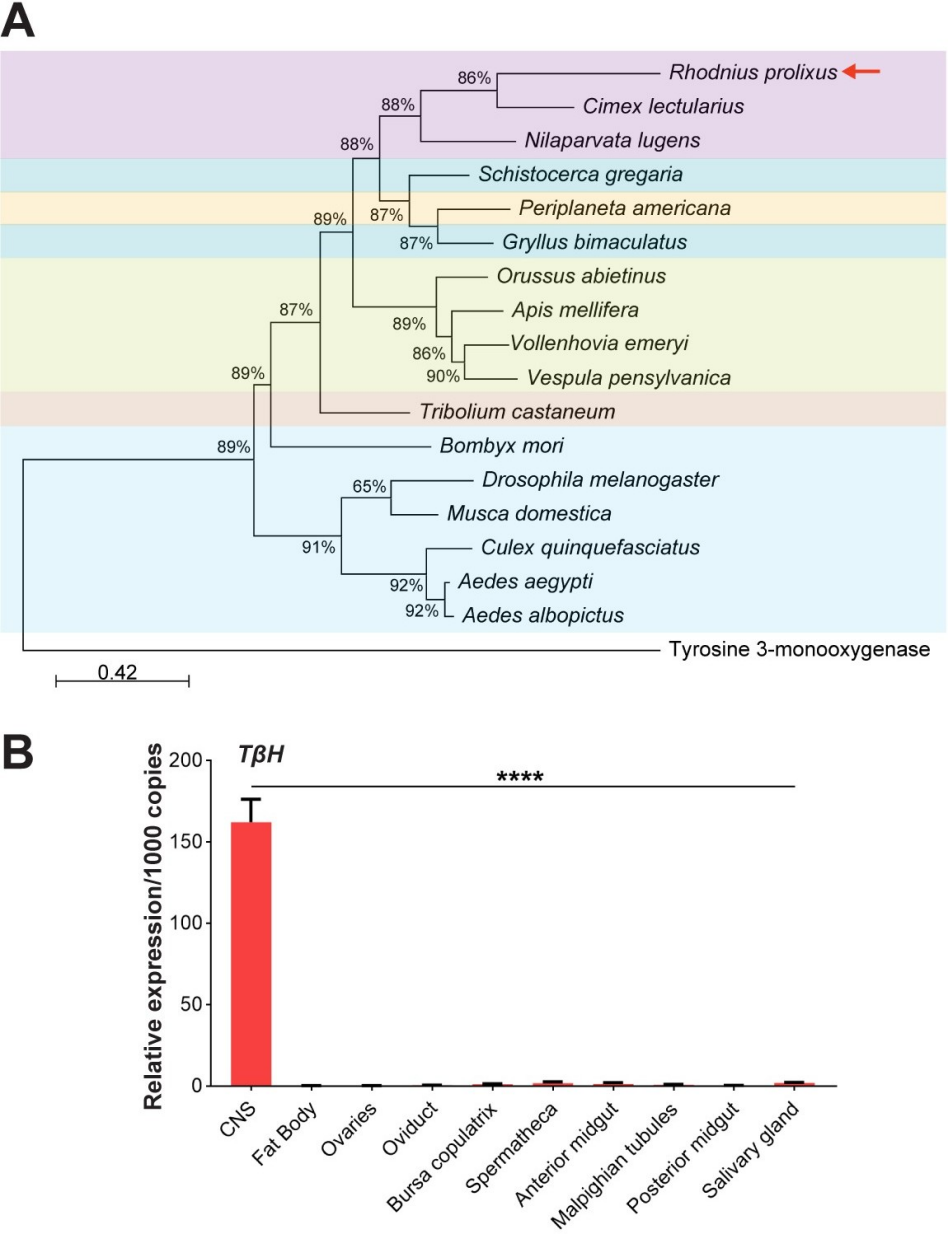
Identification of TβH and its transcript expression. (**A**) Phylogenetic relationship of TβH in insects. The evolutionary history was inferred by using the Maximum Likelihood method. The tree with the highest log likelihood (-15636.19) is shown. The tree is drawn to scale, with branch lengths measured in the number of substitutions per site. Protein sequences are labelled by species and order name. GenBank accession number: *Aedes aegypti*, AAEL010856; *Aedes albopictus*, AALC636_034546; *Apis mellifera*, NP_001071292.1; *Bombyx mori*, NP_001243923.1; *Cimex lectularius*, XP_014250928.1; *Culex quinquefasciatus*, CQUJHB002793; *Drosophila melanogaster*, FBgn0010329; *Gryllus bimaculatus*, BAO52001.1; *Musca domestica*, MDOA006509; *Nilaparvata lugens*, XP_039288853.1; *Orussus abietinus*, XP_012275259.1; *Periplaneta americana*, AFO63080.1; *Schistocerca gregaria*, XP_049845783.1; *Tribolium castaneum*, XP_974169.1; *Vespula pensylvanica*, XP_043684637.1; *Vollenhovia emeryi*, XP_011883289.1; *Rhodnius* tyrosine 3-monooxygenase like (RPRC007034)-. (**B**) Distribution of *TβH* transcript in tissues from unfed virgin female *R*. *prolixus*. RT-qPCR was used to quantify transcript levels, and data analysis was performed using the 2^−ΔCt^ method. Relative expression values on the y-axes were calculated by geometric averaging of reference genes *Rp49* and *actin*. Data are presented as mean ± SEM (n = 4–5, where each n represents a pool of tissues from 3 insects). **** p < 0.0001 (One-way ANOVA and Tukey’s test as the post hoc test).

Based on the *R*. *prolixus* genome database and using BLAST tools, we found a high-confident hit for the TβH protein sequence. A phylogenetic analysis reveals that this sequence is closely related to that from the hemipteran *Cimex lectularius*, and both are grouped with another insect of the same order, *N*. *lugens* (Fig 1A). The related sequences from other insect orders form two separate phyletic clusters, with a distribution that is consistent with the taxonomy established for the corresponding orders (Fig 1A). Tissue-specific transcript expression of *TβH* was initially investigated in unfed virgin females (Fig 1B); *TβH* transcript is almost exclusively expressed in the CNS-CC-CA, with very low expression in other tissues (Fig 1B). Although *TDC* transcript expression has a similar pattern, *TβH* mRNA levels are more than 15-fold higher than *TDC* in the CNS-CC-CA (Figs 1B and S1). The low expression of the *TDC* gene in *R*. *prolixus* was also shown by Sterkel et al., [58].

### Egg production is lower, and eggs are white in dsTβH-injected females

**Fig 2:**
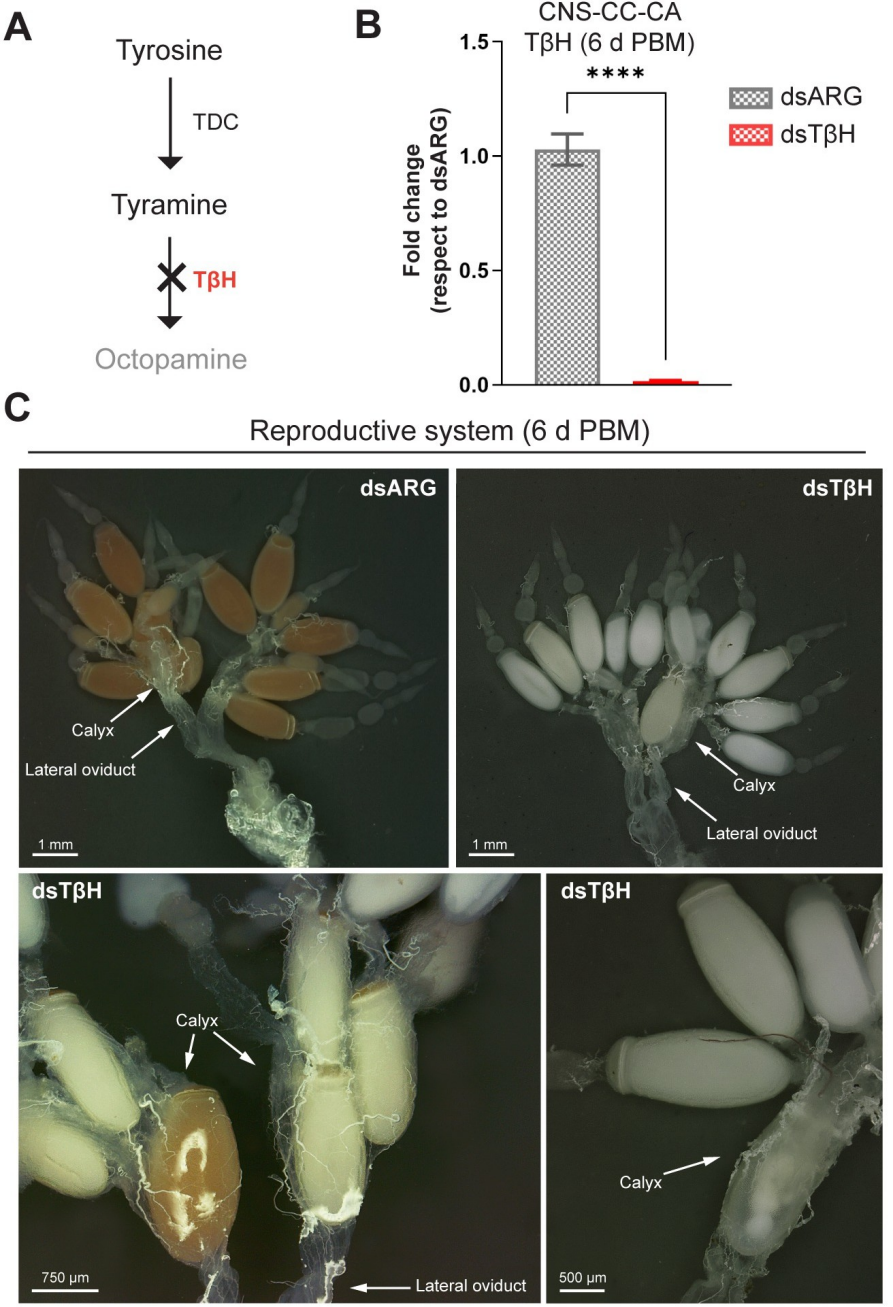
Effect of dsRNA treatment at 6 d post blood meal. **(A)** Tyrosine decarboxylase (TDC) converts tyrosine into tyramine, which is then converted to octopamine (OA) by tyramine beta-hydroxylase (TβH). *TβH* transcript downregulation is a mechanism for decreasing OA levels. **(B)** *TβH* mRNA expression in the CNS-CC-CA complex of dsTβH-injected mated females at 6 d post blood meal (6 d PBM). Transcript levels were quantified using RT-qPCR and analyzed by the 2^-ΔΔCt^ method. The y axis represents the fold change in expression relative to control (dsARG, value ∼ 1) obtained via geometric averaging using *Rp49* and *actin* as reference genes. The results are shown as the mean ± SEM (n = 7–8, where each n represents an individual tissue from 1 insect). **** p < 0.0001 (Student’s t-test). (**C**) Upper panel shows representative images of the reproductive system from dsRNA-injected insects 6 d PBM. Note the pink color of oocytes in the controls (dsARG) and white color of oocytes in the dsTβH-injected insects. Lower panels are a higher magnification of representative images which reveal eggs retained in the calyx following the knockdown of TβH. n = 10–15 females.

*TβH* transcript downregulation is a mechanism for decreasing OA levels (Fig 2A) (Monastirioti, 2003; Monastirioti et al., 1996). In order to explore *TβH* knockdown effects, we first calculated the possible off-target effects of the dsTβH. The dsRNA sequences used were compared to the *R*. *prolixus* genome using the si-Fi v21 software (default parameters). The only target sequence for dsTβH was the predicted *TβH* transcript (accession number RPRC014470; S2 Fig), with the generation of 113 potential efficient siRNA hits. After dsTβH injection, the RNAi efficiency in the CNS-CC-CA complex at 10 d PE (unfed) was more than 99% (S2 Fig). To examine the effects on egg production, dsRNA-injected females were given a blood meal at 10 d PE. Since the size of a blood meal directly influences egg production, both groups of insects (dsTβH and dsARG) were weighed after feeding and at 1 d PBM. Our results show that the blood meal size is not affected by dsRNA treatment, and neither is diuresis, indirectly evaluated as insect weight over the first 24 h PBM (S2 Fig) [49]. Next, we evaluated the ability of mated fed females to make and lay eggs. RNAi efficiency in the CNS-CC-CA at 6 d PBM was more than 99% (Fig 2B). Interestingly, the ovaries from dsTβH-injected females 6 d PBM contain smaller than normal oocytes that were white in color, with only a few chorionated, in contrast to their standard pink color in control females (Fig 2C). In addition, 6 d PBM dsTβH-injected insects had up to 3 eggs retained in each calyx which is not normally observed in controls (Fig 2C).

**Fig 3:**
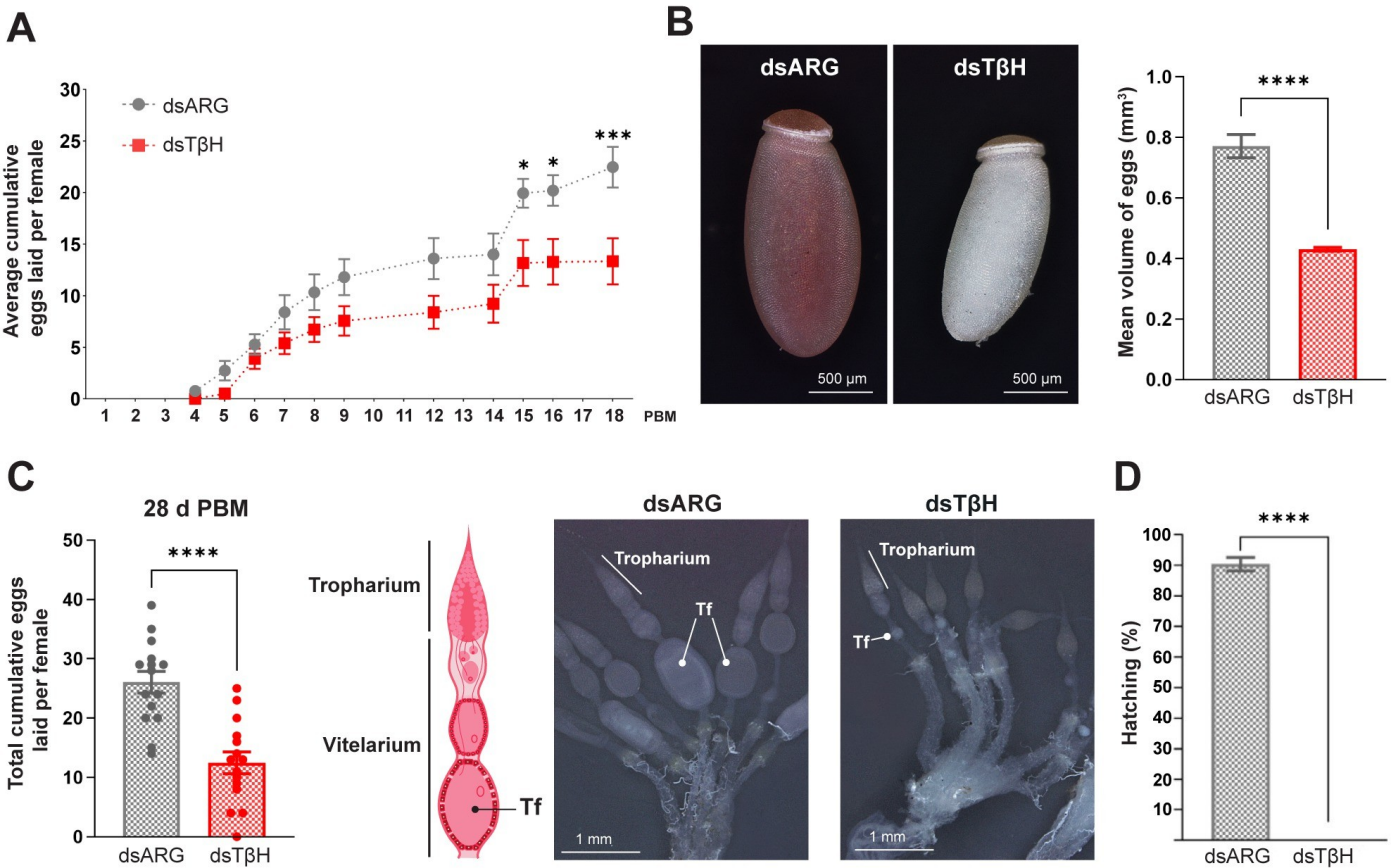
Egg production in TβH-deficient females. **(A)** Average cumulative eggs laid per mated female throughout 18 d post-blood meal (d PBM) after dsARG and dsTβH injections. The results are shown as the mean ± SEM (n = 10 to 15 females). dsARG vs. dsTβH: * p < 0.05; *** p < 0.001 (repeated-measures two-way ANOVA followed by the post-hoc Bonferroni’s test). (**B**) Representative images showing egg phenotype (left) and egg volume (right) of dsARG- and dsTβH-injected *R*. *prolixus* females. The results are shown as the mean ± SEM (n = 15 eggs). **** p < 0.0001 (Student’s t-test). (**C**) Total cumulative eggs laid per female over 28 d PBM (left) and diagram illustrating the morphology of the ovarioles and representative images showing the female reproductive system morphology (right) of dsRNA-injected females. Note the ovarioles with smaller size of tropharium and terminal oocytes after dsTβH treatment (n = 15–20 females). **** p < 0.0001 (Student’s t-test). (**D**) Percentage of hatching with respect to total eggs laid after dsRNA treatment (n = 15–20 females). **** p < 0.0001 (Student’s t-test).

Indeed, the beginning of egg laying is slightly delayed (6 ± 1 days PBM versus 5 ± 1 days PBM) and the number of eggs laid is less in dsTβH-injected females with respect to control females (Fig 3A). As expected, due to the observed ovarian phenotype (Fig 2C), the eggs laid by TβH knockdown insects are white and their volume is significantly smaller than those from control insects (Fig 3B). At the end of the first reproductive cycle (28 days PBM), each control female laid a total of ∼ 26.07 ± 1.84 eggs, while each dsTßH-injected female laid ∼ 12.47 ± 1.83 eggs (*p <* 0.0001; n = 15–20) (Fig 3C, left panel). The ovarian morphology 28 days PBM indicates smaller trophariums and terminal follicles in dsTβH-injected insects when compared to control females (Fig 3C, right panel). Most importantly, none of the white eggs laid by TβH knockdown females were viable (Fig 3D).

### TβH knockdown decreases transcript expression of JH biosynthetic enzymes and JH response genes

**Fig 4:**
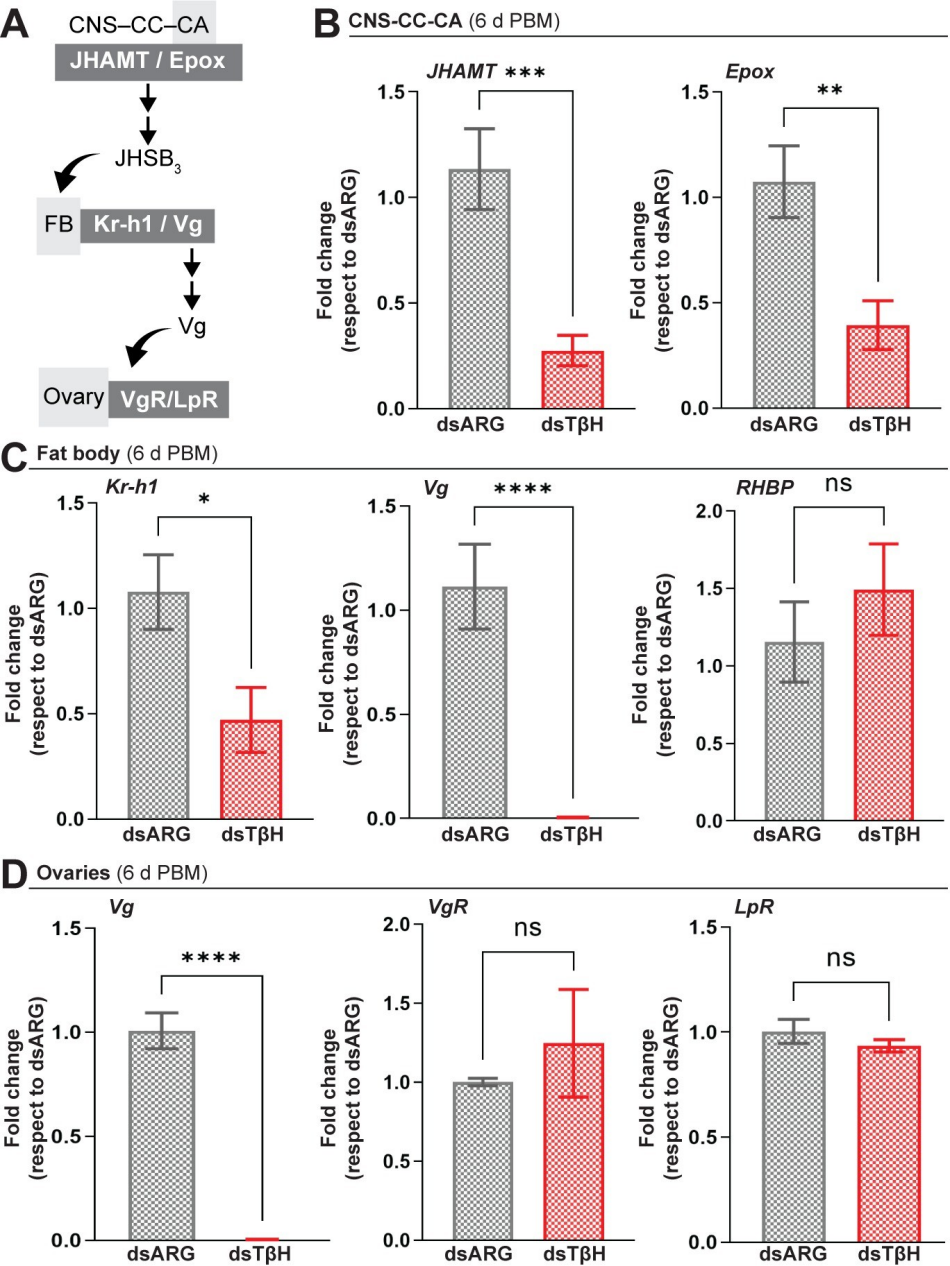
Effect of dsRNA treatment on JH biosynthetic enzymes and JH-response genes. **(A)** In the CNS-CC-CA, JHAMT and Epox are involved in JHSB_3_ production, which is released into the circulation to act on the fat body (FB). In the FB, *Kr-h1* and *Vg* are classic JH-response genes. Vg and other yolk protein precursors are then released into the circulation and accumulate in the oocytes by endocytic receptors VgR and LpR. **(B)** *JHAMT* and *Epox* mRNA expression in the CNS-CC-CA complex is decreased in dsTβH-injected mated females at 6 d post blood meal (6 d PBM). **(C)** *Kr-h1*, *Vg* and *RHBP* mRNA expression in the fat body of dsARG- and dsTβH-injected mated females. Note that *Kr-h1* and *Vg* expression is decreased in dsTβH-injected females. **(D)** *Vg*, *VgR* and *LpR* mRNA expression in the ovaries of dsARG- and dsTβH-injected mated females. dsTβH injection results in a reduction in *Vg* expression. Transcript levels were quantified using RT-qPCR and analyzed by the 2^-ΔΔCt^ method. For C and D, the y axes represent the fold change in expression relative to control (dsARG, value ∼ 1) obtained via geometric averaging using *Rp49* and *actin* as reference genes. The results are shown as the mean ± SEM (n = 7–8, where each n represents an individual tissue from 1 insect). * p < 0.05; ** p < 0.01; *** p < 0.001; **** p < 0.0001; ns, not significant (Student’s t-test). Epox, methyl farneseoate epoxidase. JHAMT, juvenile hormone acid O-methyltransferase; Kr-h1, Krüppel homolog 1; LpR, lipophorin receptor; RHBP, *Rhodnius* heme-binding protein; Vg, vitellogenin; VgR, vitellogenin receptor.

To evaluate the possible molecular mechanism responsible for the phenotypes observed in dsTβH-injected females, we analyzed the expression of JH biosynthetic enzymes and JH response genes. Juvenile hormone acid methyltransferase (JHAMT) and methyl farneseoate epoxidase (Epox) are enzymes involved in JH production (Fig 4A) (Noriega, 2014). In *R*. *prolixus*, *JHAMT* and *Epox* mRNA expression has been correlated with the patterns of JH synthesis [33]. At 6 d PBM, mRNA levels for both enzymes in the CNS-CC-CA are significantly lower in dsTβH-injected females when compared to controls (*p* < 0.001 and *p* < 0.01, respectively; Fig 4B).

Recently, we reported that JHSB_3_ signaling, through Met and Tai, modulates transcript expression of downstream genes, such as *Kr-h1* and *Vg* in the fat body (Fig 4A) [41]. Although Vg is the main YPP, *Rhodnius* heme-binding protein (RHBP), which generates the characteristic pink coloration of eggs, is also described as a YPP of critical importance [40]. RHBP seems to be regulated by JHSB_3_ but not to the same extent as Vg [41]. Here, we show that at 6 d PBM mRNA expression of *Kr-h1* and *Vg* are significantly downregulated in the fat body of dsTβH-injected females with respect to controls (*p* < 0.05 and *p* < 0.0001, respectively; n = 7–8), while *RHBP* transcript levels appear to not be influenced by *TβH* downregulation (Fig 4C). *R*. *prolixus* follicle cells themselves can also synthesize Vg under JH control [41]; *Vg* expression is also significantly downregulated in the ovaries of dsTβH-injected females (*p* < 0.0001; n = 7–8) while the *vitellogenin receptor* (VgR) and the *lipophorin receptor* (LpR) transcript expression, endocytic receptors involved in YPP uptake (Fig 4A), are not affected (Fig 4D).

**Fig 5:**
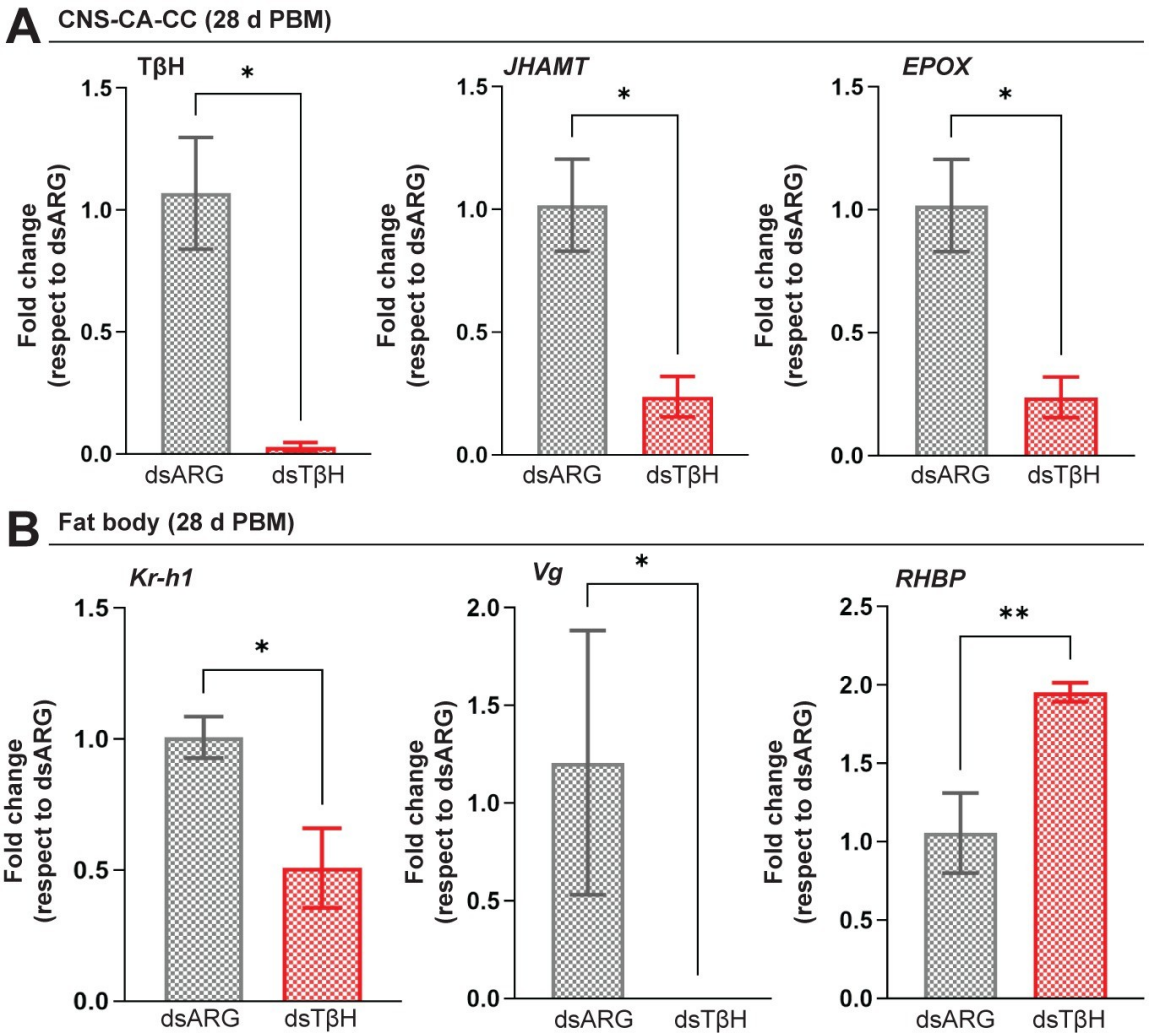
Effect of dsRNA treatment on JH biosynthetic enzymes and JH-response genes 28 days post blood meal (d PBM). **(A)** *TβH*, *JHAMT* and *Epox* mRNA expression in the CNS-CC-CA complex of dsARG- and dsTβH-injected females at 28 days post blood meal (28 d PBM). **(B)** *Kr-h1*, *Vg* and *RHBP* mRNA expression in the fat body of dsARG- and dsTβH-injected insects. RT-qPCR was used to quantify transcript levels, and data analysis was performed using the 2^−ΔΔCt^ method. Relative expression values on the y-axes represent the fold change in expression relative to control (dsARG, value ∼ 1) and were calculated by geometric averaging of reference genes *Rp49* and *actin*. Data are presented as mean ± SEM (n = 7–8, where each n represents an individual tissue from 1 insect). * p < 0.05; ** p < 0.01 (Student’s t-test). CNS-CC-CA, central nervous system-corpora cardiaca-corpora allata; Epox, methyl farneseoate epoxidase; JHAMT, juvenile hormone acid O-methyltransferase; Kr-h1, Krüppel homolog 1; RHBP, *Rhodnius* heme-binding protein; tyramine β-hydroxylase (TβH); Vg, vitellogenin.

At 28 d PBM, with an RNAi efficiency around 99%, the statistically significant downregulation of *JHAMT* and *Epox* mRNA in the CNS-CC-CA (Fig 5), as well as *Kr-h1* and *Vg* in the fat body (Fig 5B) of dsTβH-injected insects is still present. Interestingly, at this time point, *RHBP* transcript levels are now significantly increased in the fat body of *TβH*-injected females (*p* < 0.01; n = 7–8, Fig 5B).

### TβH knockdown decreases Vg protein expression in the fat body and decreases YPP accumulation by the ovaries

**Fig 6:**
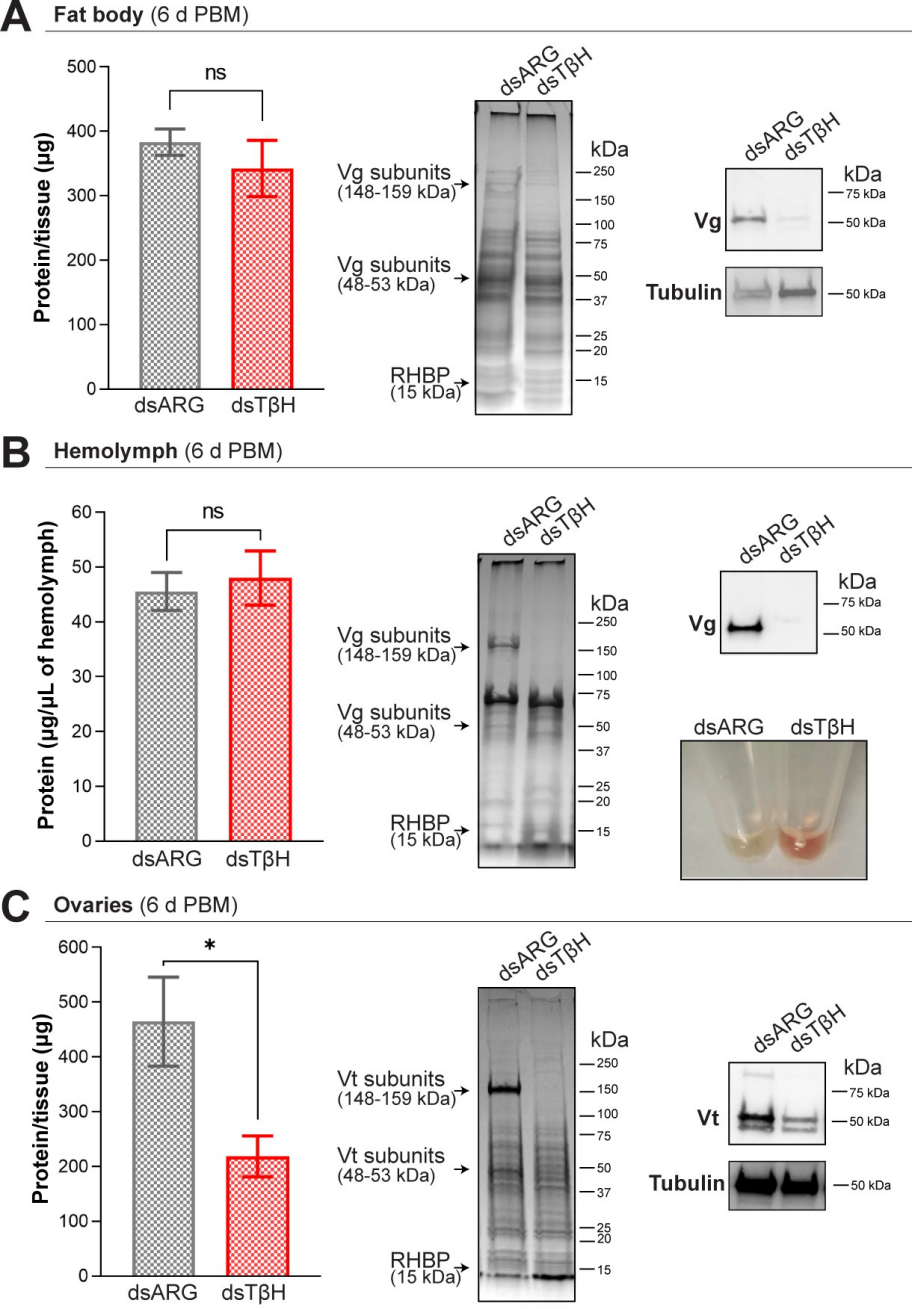
Effect of dsRNA treatment on yolk protein precursors 6 days post blood meal (d PBM). **(A)** Total protein content per fat body (mean ± SEM; n = 5–6, where each n represents tissue from 1 insect), SDS-PAGE analysis (10 μg/line, images representative of 3 independent experiments), and western blot (5 μg/line; images representative of 3 independent experiments) after *TβH* knockdown. **(B)** Total protein per μL in the hemolymph (mean ± SEM; n = 7–8, where each n represents hemolymph from 1 insect), SDS-PAGE analysis (5 μg/line, images representative of 3 independent experiments), western blot (1 μg/line; images representative of 3 independent experiments), and color of the hemolymph after knockdown of *TβH* (images representative of n = 5). (**C)** Total protein content per ovary (mean ± SEM; n = 5–6, where each n represents tissue from 1 insect), SDS-PAGE analysis (10 μg/line, images representative of 3 independent experiments) and western blot (5 μg/line; images representative of 3 independent experiments) after knockdown of *TβH*. ** *p* < 0.01; ns, not significant (Student’s t-test). In the SDS-PAGE analyses the molecular weights of the main yolk protein precursor, Vg (or Vt in the ovaries) and RHBP are indicated. RHBP, *Rhodnius* heme-binding protein; Vg, vitellogenin; Vt, vitellin.

The total protein content in the fat body at 6 d PBM is comparable in both dsARG- and dsTβH-injected insects, with a slight but non-significant decrease in the latter (dsARG vs. dsTβH: 384.2 ± 20.57 μg/fat body vs. 343.6 ± 43.49 μg/fat body, n = 5–6, *p* < 0.05); Fig 6A, left panel). SDS-PAGE analysis and Coomassie blue staining show less high molecular weight proteins in dsTβH-injected insects (10 μg of protein/lane; Fig 6A, middle panel) and western blot reveals a significant decrease in Vg protein expression (1 μg of protein/lane; Fig 6A, right panel). The total protein circulating in the hemolymph has a similar pattern to the fat body; that is, no significant quantitative changes between groups (dsARG vs. dsTβH: 45.58 ± 3.459 μg/μl of hemolymph vs. 48.03 ± 4.96 μg/μl of hemolymph, n = 7–8; Fig 6B, right panel). However, the differences in circulating protein in dsTβH-injected insects according to their molecular weights are clearer, with less high molecular weight proteins. Thus, in dsTβH-injected insects there is a decrease in Vg protein expression, detected by SDS-PAGE analysis as well as western blot (Fig 6B, middle and upper left panel), but the expression of low molecular weight proteins (among which is RHBP) increases. In addition, whereas control insects have a yellowish transparent hemolymph, higher concentrations of RHBP are obvious by the reddish hemolymph extracted from dsTβH-injected insects (Fig 6B, lower right panel). In addition, the ovaries from TβH knockdown insects only accumulate around 50% of the total protein normally found in the ovaries of control females (dsARG vs. dsTβH: 464.6 ± 81.19 μg/ovaries vs. 216.2 ± 23.86 μg/ovaries, n = 5–6; Fig 6C, left panel). In particular, the accumulation of the main YPP, vitellin, is severely compromised, as seen by SDS-PAGE analysis and western blot (Fig 6C, middle and right panels, respectively). Full-length blots are presented in S3 Fig.

### OA modulates transcript expression of JH biosynthetic enzymes and JH-response genes

**Fig 7:**
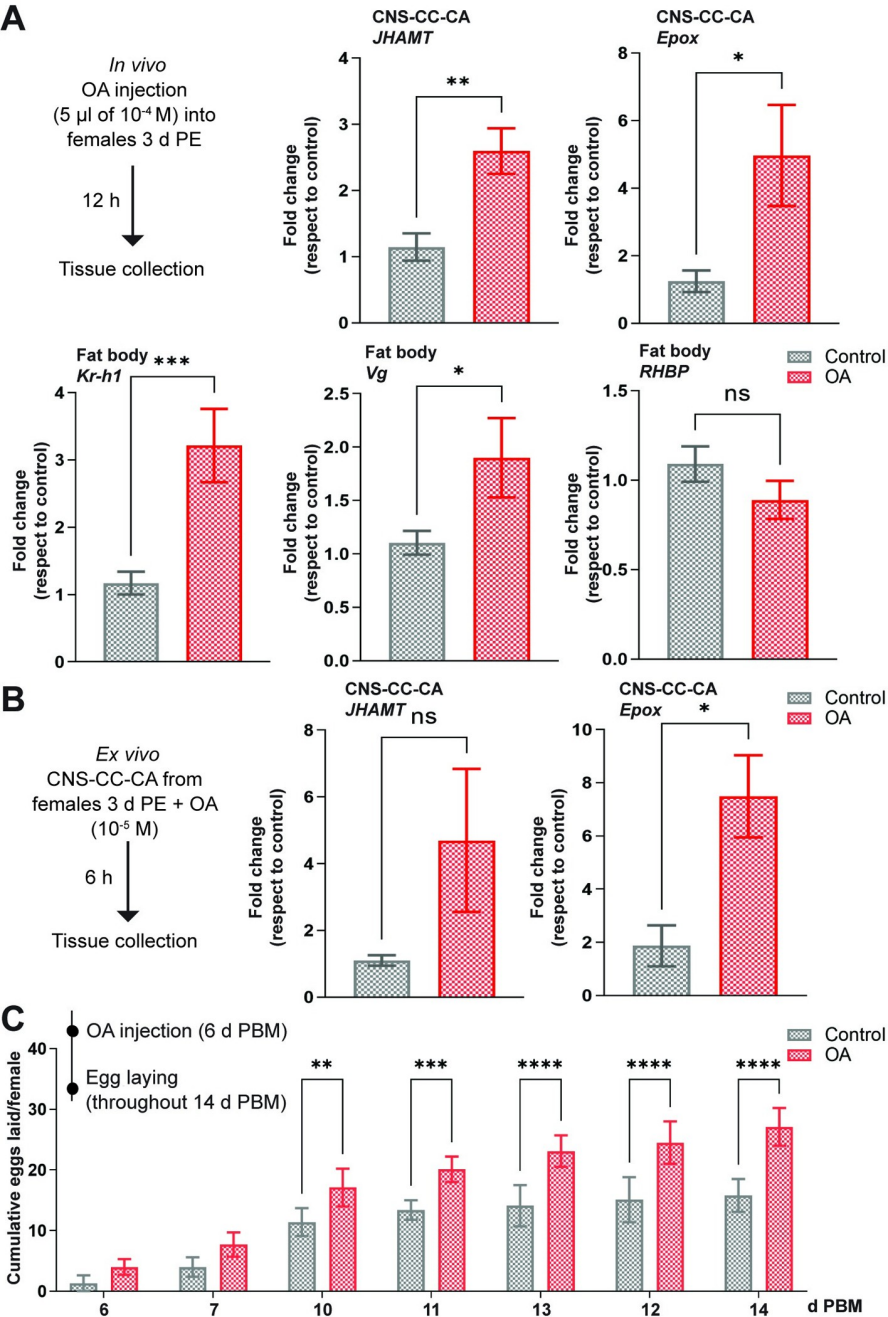
Effect of exogenous octopamine (OA) treatment. **(A)** In vivo assays: OA (5 μL of saline containing 10^−4^ M OA) was injected into unfed virgin females 3 days post ecdysis (d PE), and transcript levels for *JHAMT* and *Epox* in the CNS-CC-CA and *Kr-h1*, *Vg* and *RHBP* in the fat body were measured 12 h later. All transcripts were up-regulated by OA except for RHBP. The results are shown as the mean ± SEM (n = 5–6, where each n represents an individual tissue from 1 insect). Transcript expression was quantified using RT-qPCR and analyzed the 2^−ΔΔCt^ method. **(B)** Ex vivo assays: OA was added to the incubation medium (final concentration: 10^−5^ M) containing a pool of 3 tissues (CNS-CC-CA from virgin females 3 d PE). After 6 h, *JHAMT* and *Epox* mRNA expression was evaluated. The results are shown as the mean ± SEM (n = 4–5, where each n represents a pool of 3 tissues). For A and B, the y axes represent fold change in expression relative to control (saline, value ∼1) obtained via geometric averaging using *Rp49* and *actin* as reference genes. ns, not significant; * p < 0.05; ** p < 0.01; *** p < 0.001 (Student’s t-test). **(C)** OA treatment on egg laying. OA (5 μL of saline containing 10^−4^ M of OA) was injected into fed mated females 6 days post blood meal (d PBM), and egg laying pattern was recorded throughout 14 d PBM. The results are shown as the mean ± SEM (n = 10–15 females). ** p < 0.01; *** p < 0.001; **** p < 0.0001 (repeated-measures two-way ANOVA followed by the post-hoc Bonferroni’s test). CNS-CC-CA, central nervous system-corpora cardiaca-corpora allata; Epox, methyl farneseoate epoxidase; JHAMT, juvenile hormone acid O-methyltransferase; Kr-h1, Krüppel homolog 1; OA, octopamine; RHBP, *Rhodnius* heme-binding protein; Vg, vitellogenin.

To test whether transcript expression of JH biosynthetic enzymes and JH-response genes (Fig 7A) are influenced by OA, we injected unfed adult virgin females 3 d PE with OA (5 μL of 10^−4^ M) or 5 uL of saline (control) and 12 h later dissected the CNS-CC-CA and the fat body for qPCR (Fig 7A). *JHAMT* and *Epox* transcript expression are both upregulated in the CNS-CC-CA after OA injection (Fig 7A, *p* < 0.01 and *p* < 0.05, respectively; n = 5–6) as are the JH-response gene *Kr-h1* and *Vg* in the fat body (Fig 6A, *p* < 0.001 and *p* < 0.05, respectively; n = 4–5). As shown when *TβH* mRNA is downregulated, *RHBP* transcript levels in the fat body are not significantly influenced by OA injection (Fig 7A, *p* > 0.05; n = 5–6). When the CNS-CC-CA from 3 d PE unfed virgin females are incubated ex vivo in 10^−5^ M OA, an increase in *JHAMT* and *Epox* mRNA is also observed (Fig 7B, *p* > 0.05 and *p* < 0.05, respectively; n = 4–5).

In addition, injection of OA (5 μL of 10^−4^ M) into 6 d PBM mated females, a time when egg laying is just beginning, significantly increases the eggs laid per female by 14 d PBM (Fig 7C).

## Discussion

The kissing bug, *R*. *prolixus*, is a medically important blood-feeding insect, being a primary vector of the parasite *T*. *cruzi*, the causative agent of Chagas disease [38, 39]. Understanding its reproductive biology is of some relevance not only from a scientific perspective, but also in the medical context for controlling the population and thereby spread of the disease. Here, we demonstrate that the downregulation of the enzyme TβH, impairs successful reproduction at least in part, by decreasing the expression of enzymes involved in JH biosynthesis, and thereby interfering with oogenesis, ovulation and oviposition. It is important to highlight that we did not directly measure the decrease in OA titers. However, we strongly suggest that when TβH is downregulated, OA levels decrease, as demonstrated in the dipteran *D*. *melanogaster*, coleopteran *T*. *castaneum*, hemipteran *N*. *lugens*, and lepidopteran *M*. *separata* [27, 45–48].

OA is synthesized in neurons in the brain, but also typically in dorsal and ventral unpaired median neurons throughout ganglia of the ventral nerve cord [3]. Therefore, TβH is predominantly localized to the nervous system in insects [47, 59, 60], which is confirmed with our findings of an almost exclusive *TβH* mRNA expression in the CNS. Neurons containing OA have axons that project to neurohemal sites but also directly to peripheral tissues, with terminals on targets such as the reproductive system [7]. The effects of OA on these target tissues are varied depending on the type of OA receptor present. In the African migratory locust *Locusta migratoria*, octopaminergic neurons innervate the oviducts and inhibit muscle contraction, thus influencing oviposition [16]. Phentolamine, a receptor antagonist, effectively inhibited the effects of octopamine, indicating that its mode of action likely involves α-adrenergic receptors [16, 61]. In *R*. *prolixus*, not only OA but also TA play a role in relaxing the oviducts, likely acting through OAβ2R and Tyr1-R receptors [43]. thereby facilitating ovulation. Furthermore, in the bursa, these biogenic amines may contribute to egg retention by reducing bursal contractions and inhibiting oviposition [42]. In *D*. *melanogaster*, octopaminergic neurons also innervate the female reproductive system, regulating oogenesis, ovulation, egg movement, and sperm storage by acting on OAα1R and OAβ2R [7]. As mentioned earlier, OA can also act as a neurohormone and therefore its release into the hemolymph can affect multiple tissues, acting through specific receptors. Therefore, the overall affect of OA on reproductive processes is complex, as noted in a variety of reviews [3, 7, 16, 19].

In *R*. *prolixus* females, a blood meal is the main stimulus for egg production [19, 37]. Additionally, the blood meal serves as a signal to stimulate the synthesis and release of greater amounts of JH into the circulation [33]. Following feeding, two primary functions of circulating JH include promoting the synthesis of YPPs in the fat body and opening intercellular spaces in the ovarian follicular epithelium allowing uptake of YPP into the growing oocyte (patency) [19, 37]. Here, we demonstrate that both JH-response genes *Kr-h1* and *Vg* mRNA expression are significantly reduced by the downregulation of *TβH*, a scenario also observed when the JH signaling pathway is impaired [41]. The hypothesis that OA may help regulate JH titers becomes more evident as we demonstrate that a reduction of *TβH* mRNA decreases the expression of two essential enzymes associated with JH production, *JHAMT* and *Epox*, and that OA stimulates their expression during in vivo and ex vivo experiments. In *Diploptera punctata* females, OA was identified within the CA as a neuromodulator of JH production. Thus, exogenous OA inhibits JH biosynthesis by the CA and also results in a pronounced hyperpolarization of CA cells. This latter effect has been shown to occur with electrical stimulation of the axon tracts innervating the CA. It is possible that OA acts directly on the *D*. *punctata* CA cells but also indirectly by influencing the release of the inhibitory neuropeptide, allatostatin, from their terminals within the CA [21]. In *Gryllus bimaculatus* females, the ratio of the amount of OA to that of its metabolite in the CA indicates that OA is released and metabolized from nerve terminals in this gland [62]. There, OA appears to inhibit JH production via calcium signaling [22]. In *L*. *migratoria* OA stimulates JH release from the CA via octopaminergic receptors coupled to adenylate cyclase [23]. OA would appear to regulate JH production in a species-specific manner. Recently, Finetti et al. [44] showed that silencing OAα1R in *R*. *prolixus* females does not affect the expression of JH-response genes, indicating that this receptor is not involved in aspects of JH production; however other OA receptors may be. Further investigation is required to identify which of the remaining four GPCRs are specifically expressed in the CA and what role they play in the regulation of JH biosynthesis.

In dsTβH-injected insects, blood digestion appears unaffected, allowing blood components like amino acids, lipids, and heme molecules to cross the digestive barrier and reach the fat body for storage or YPP biosynthesis. YPPs dependent on JH signaling, such as Vg, show a profound reduction in *TβH* knockdown females. However, the minimal difference in total protein content observed in the fat body of dsARG- and dsTβH-injected insects supports the notion that not all YPPs are strictly regulated by JH signaling. Those of low molecular weight appear to use the developed biosynthetic machinery and nutrient availability in the fat body to enhance their production, potentially compensating for the absence of Vg. The pink-like color of the hemolymph in dsTβH-injected females indicates an accumulation of YPPs in the hemolymph (particularly RHBP) due to either an increase in their synthesis or a reduced uptake into oocytes or both. The predicted lower JH titers in dsTβH-injected females also may suggest reduced patency. This is substantiated by the low protein content in the ovaries, the presence of white eggs (indicative of impaired RHBP uptake), and the decrease in total cumulative eggs produced per female throughout the reproductive cycle. A reduction in *TβH* mRNA expression in mated *N*. *lugens* females leads to a decrease in ovarian and fat body protein content, along with diminished Vg expression at both protein and transcript levels. This depletion affects processes such as oogenesis and egg laying, and consequently results in a reduced number of offspring in *N*. *lugens* [26, 27]. Importantly, in addition to OA modulating JH biosynthesis, our results and those found in *N*. *lugens* suggest that a direct effect of OA on the fat body in modifying Vg expression cannot be ruled out. Further experiments are needed to clarify this. It is worth mentioning that TA titers may increase in dsTBH-treated insects due to less TA being synthesized into OA. However, the decrease in Vg expression is more likely due to a lack of OA, since we recently demonstrated that TA increases Vg titers [53]. Previously, we suggested that OA influences oviposition in *R*. *prolixus* since OA reduces oviduct contraction amplitude by binding to OAβ2R, leading to elevated cAMP levels and muscle relaxation [42, 43]. Our current study reaffirms OA’s role during egg movement, particularly at ovulation, since dsTβH-treated insects retain numerous eggs within the calyx. Furthermore, a single injection of OA into fed and mated *R*. *prolixus* females accelerates egg laying, thus confirming the facilitative role of OA in egg laying. OA can also bind to some TA receptors [6]; thus, the effects shown here could be due to OA’s action on specific OA GPCRs but also on TA GPCRs, since both amines have been suggested to participate in egg production and egg laying [43, 44]. In *D*. *melanogaster*, TβH mutant flies lacking only OA (TA is still present) are reproductively sterile, at least in part because OA regulates ovary and oviduct contractions that enable the movement of eggs during ovulation and oviposition [59, 63, 64]. Treatment with OA also increases the number of eggs laid by females of the Indian meal moth, *Plodia interpunctella* [65], the rice leaf bug *Trigonotylus caelestialium* [17], the diamondback moth, *Plutella xylostella* [66], and the dark black chafer *Holotrichia parallela* [20]. On the other hand, OA decreases the number of eggs laid in the western tarnished plant bug *Lygus hesperus* [18] but has no effect on oviposition in the corn earworm *Helicoverpa zea* [15].

It should be emphasised here that since OA is a neurotransmitter, neuromodulator and neurohormone in insects [1] its precise sites of action in influencing JH titers, oogenesis, ovulation, and egg laying are not yet fully identified. Thus, the impact of OA signaling on insect reproduction merits further investigation, particularly since OA signaling holds the potential to be a target for the development of innovative insecticides with unique modes of action. Notably, OA receptors stand out as the only commercially targeted receptors among biogenic amines [67]. This study offers valuable insights into a key gene within the OA biosynthesis pathway, crucial for successful reproduction in the medically important insect *R*. *prolixus*, thereby contributing to the potential development of novel approaches for insect pest management. For example, targeting specific genes for symbiont-mediated RNAi is a powerful technology with potential for biocontrol against tropical disease vectors. Identification of specific targets for these new types of insecticides, could result in inhibiting egg production and reducing disease expansion. This approach has already been tested in *R*. *prolixus* [68].

## Supporting information

S1 Fig *Tyrosine decarboxylase* (*TDC)* transcript expression. Distribution of *TDC* transcript in unfed adult female *R*. *prolixus*. The transcript levels were quantified using RT-qPCR and analyzed by the 2^−ΔCt^ method. The y-axes represent the relative expression obtained via geometric averaging using *Rp49* and *actin* as reference genes. The results are shown as the mean ± SEM (n = 4–5, where each n represents a pool of tissues from 3 insects). **** p < 0.0001 (One-way ANOVA and Tukey’s test as the post hoc test).
(TIF)

S2 Fig Design of dsRNA and some physiological effects on TβH-deficient females. (A) Graphical output of the predicted target sequence and efficient siRNA hits of one of the TβH fragment used for the synthesis of dsRNA calculated by the si-Fi v21 software (program designed for RNAi off-target analysis and silencing efficiency predictions). The only target sequence found by the software was the one predicted to be *R*. *prolixus* TβH enzyme (accession number: RPRC014470). The program also calculated the possible number of efficient siRNAs hits (113) based upon the contextual similarity among sequences using *R*. *prolixus* genome. (B) At 3 days post ecdysis (d PE), adult females were injected with 5 μL saline containing 5 μg of dsTβH or dsARG. Seven days later, insects were dissected (before a blood meal) and dsRNA efficiency was tested by RT-qPCR. After confirming transcript downregulation, dsARG and dsTβH-injected females were weighed, fed and reweighed to measure any change in feeding behavior (recently fed) or diuresis rate (1 day post blood meal, d PBM). ns, not significant; **** p < 0.0001 (Student’s t-test).
(TIF)

S3 Fig Effect of dsRNA treatment on yolk protein precursors. (A) The uncropped images for western blots (5 μg/line; images representative of 3–5 independent experiments) shown in Fig 5. Blots were probed first with the anti-Vg antibody; after stripping with RestoreTM PLUS Western blot Stripping buffer (Thermo Fisher Scientific, Mississauga, ON, Canada), the blots were then re-probed with the anti-tubulin antibody. He, hemolymph; Vg, vitellogenin.
(TIF)

S1 Table Primers used for qPCR and dsRNA synthesis. (DOCX)

S1 Raw image (PDF)
