# Supplementary figures and images for: Octopamine is required for successful reproduction in the classical insect model, Rhodnius prolixus
Source: PLoS One. 2024 Jul 12;19(7):e0306611. doi: 10.1371/journal.pone.0306611 (PMC11244822; doi:10.1371/journal.pone.0306611)

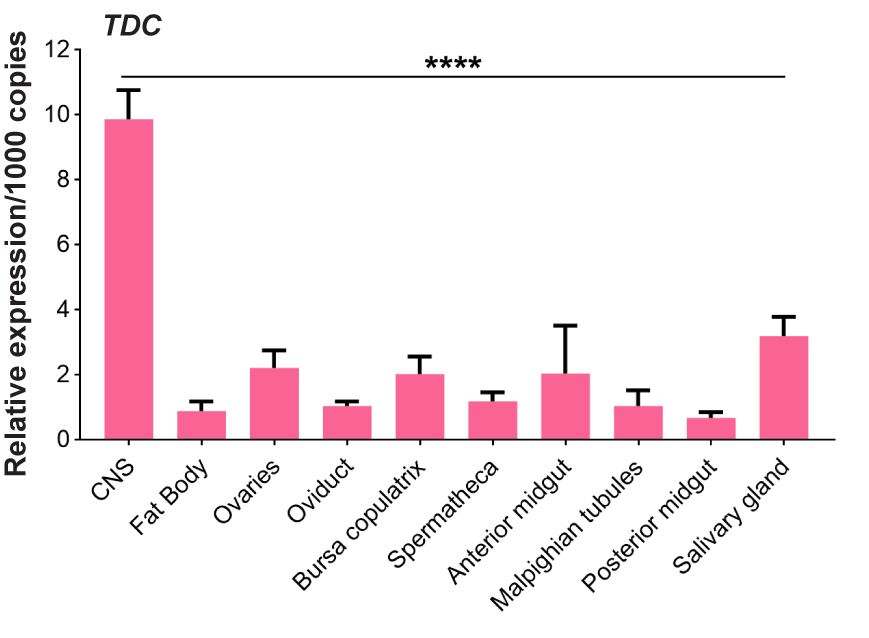

Supplement: S1 Fig — Distribution of TDC transcript in unfed adult female R. prolixus. The transcript levels were quantified using RT-qPCR and analyzed by the 2−ΔCt method. The y-axes represent the relative expression obtained via geometric averaging using Rp49 and actin as reference genes. The results are shown as the mean ± SEM (n = 4–5, where each n represents a pool of tissues from 3 insects). **** p < 0.0001 (One-way ANOVA and Tukey’s test as the post hoc test). (TIF) [file pone.0306611.s001.tif]

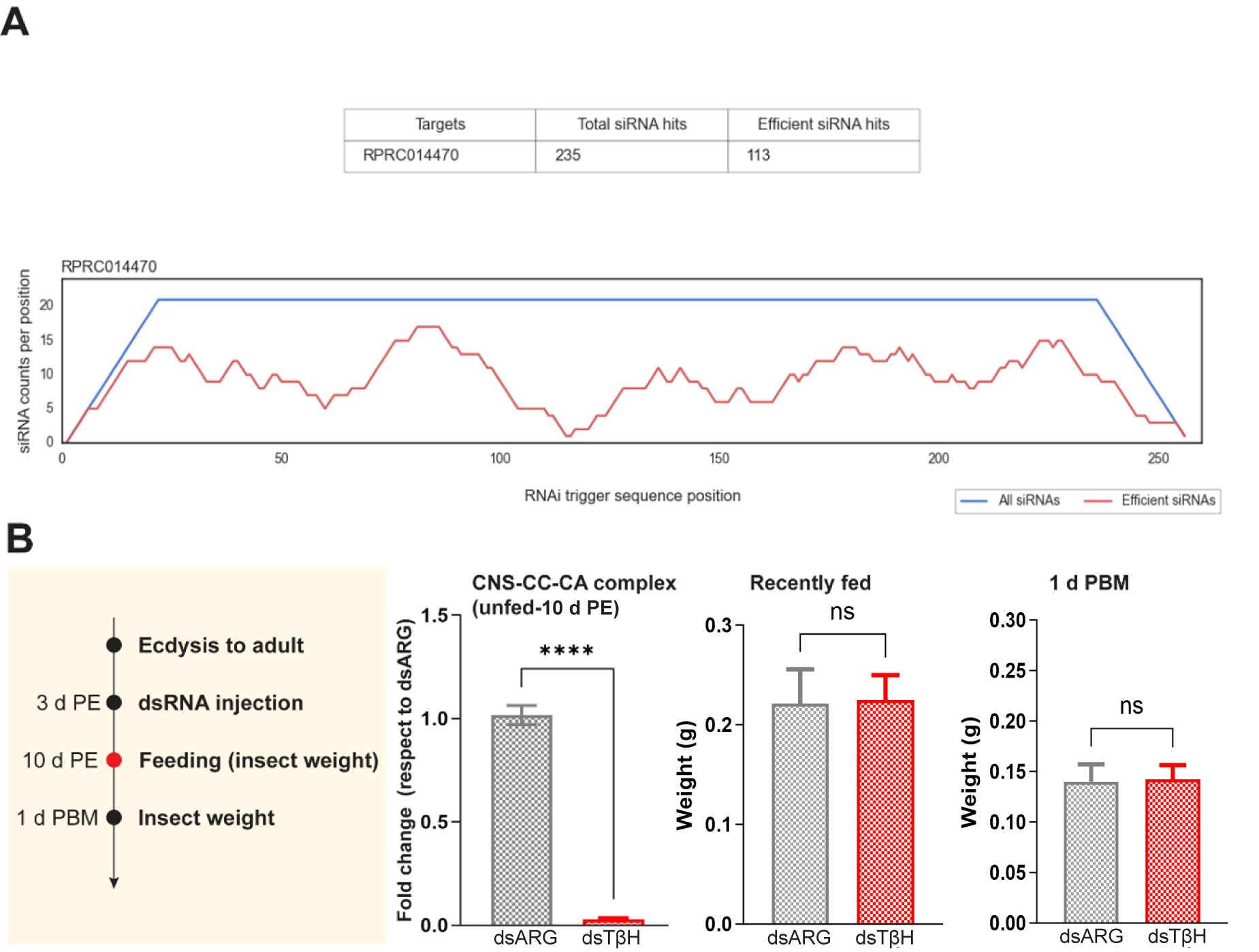

Supplement: S2 Fig — (A) Graphical output of the predicted target sequence and efficient siRNA hits of one of the TβH fragment used for the synthesis of dsRNA calculated by the si-Fi v21 software (program designed for RNAi off-target analysis and silencing efficiency predictions). The only target sequence found by the software was the one predicted to be R. prolixus TβH enzyme (accession number: RPRC014470). The program also calculated the possible number of efficient siRNAs hits (113) based upon the contextual similarity among sequences using R. prolixus genome. (B) At 3 days post ecdysis (d PE), adult females were injected with 5 μL saline containing 5 μg of dsTβH or dsARG. Seven days later, insects were dissected (before a blood meal) and dsRNA efficiency was tested by RT-qPCR. After confirming transcript downregulation, dsARG and dsTβH-injected females were weighed, fed and reweighed to measure any change in feeding behavior (recently fed) or diuresis rate (1 day post blood meal, d PBM). ns, not significant; **** p < 0.0001 (Student’s t-test). (TIF) [file pone.0306611.s002.tif]

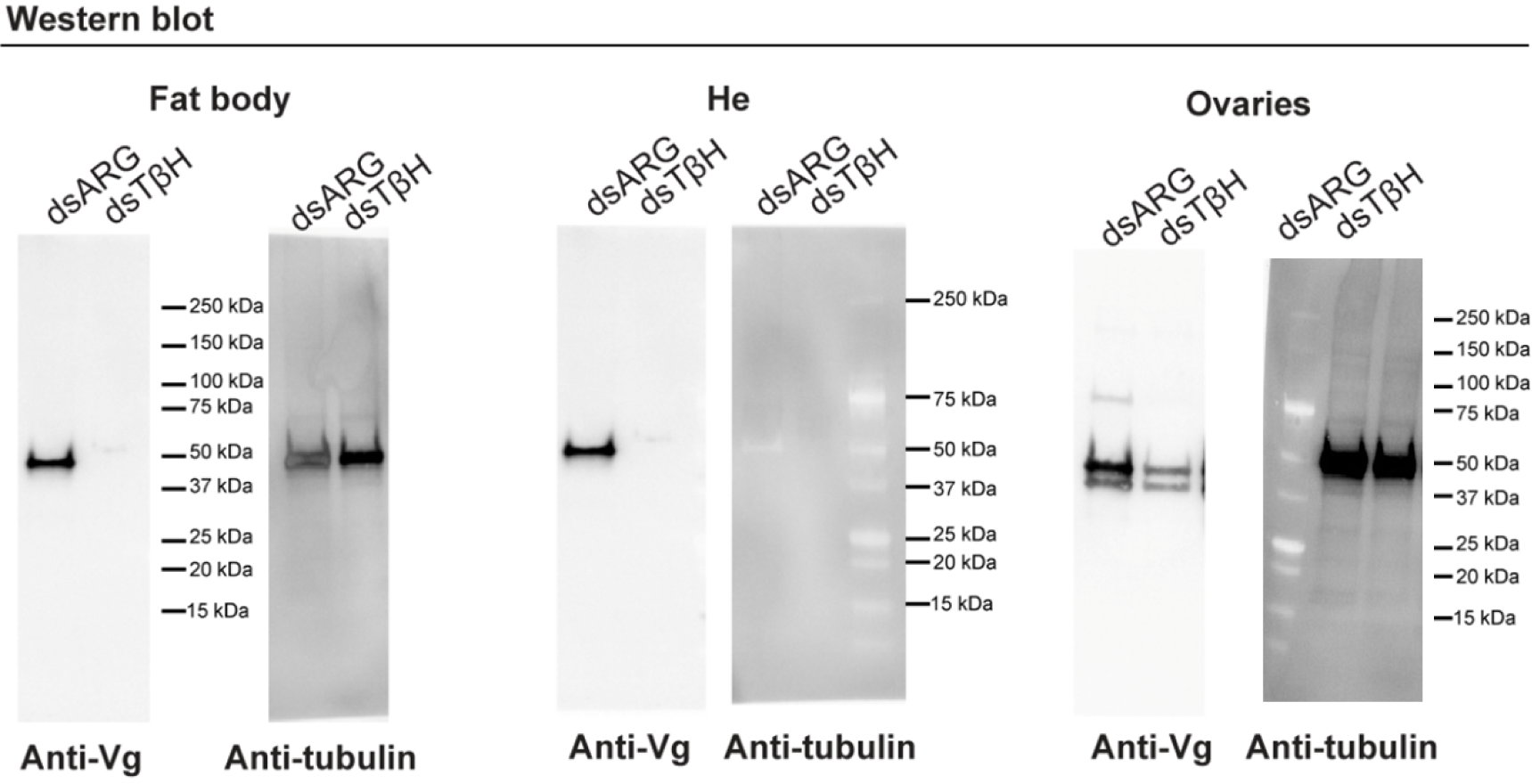

Supplement: S3 Fig — (A) The uncropped images for western blots (5 μg/line; images representative of 3–5 independent experiments) shown in Fig 5. Blots were probed first with the anti-Vg antibody; after stripping with RestoreTM PLUS Western blot Stripping buffer (Thermo Fisher Scientific, Mississauga, ON, Canada), the blots were then re-probed with the anti-tubulin antibody. He, hemolymph; Vg, vitellogenin. (TIF) [file pone.0306611.s003.tif]
