# Supplementary material for: Octopamine is required for successful reproduction in the classical insect model, Rhodnius prolixus
Source: PLoS One. 2024 Jul 12;19(7):e0306611. doi: 10.1371/journal.pone.0306611 (PMC11244822; doi:10.1371/journal.pone.0306611)
Supplement: S1 Table — (DOCX) [file pone.0306611.s004.docx]

| **Gene code** | **Primers to qPCR** | **Sequence (5🡪3)** |
| --- | --- | --- |
| **qPCR** | | |
| RPRC014470 | TβH_forward | GGCGTCATTGAACTTGGTTT |
|  | TβH _reverse | AAGCTGCCGACGTACATTCT |
| RPRC011470 | TDC_forward | GGCACCTCAACAACCAGAAT |
|  | TDC_reverse | GAAGGGTACGAATTGCCTGA |
| RPRC014398 | Kr-h1_forward | ACAACCTGTAGTGGCTGTCG |
|  | Kr-h1_reverse | CGTACACTGTAGCGTGTCGT |
| RPRC013511 | Vg 1_forward | TTGCTAGTCGCATGAACCTG |
|  | Vg 1 _reverse | TTTAGTGGTGCATCGCTCTG |
| RPRC009875 | Actin_forward | AGAGAAAAGATGACGCAGATAATGT |
|  | Actin _reverse | ATATCCCTAACAATTTCACGTTCG |
| RPRC014419 | Rp49_forward | GTGAAACTCAGGAGAAATTGGC |
|  | Rp49_reverse | AGGACACACCATGCGCTATC |
| RPRC011659 | JHAMT_forward | GGACCAGGCGATGTTACTTT |
|  | JHAMT_reverse | CCAAATCATCAGAAATATCGCTTCC |
| RPRC000513 | Epox_forward | CGGAGAATTGATTCATGATGATTGG |
|  | Epox_reverse | GTAACGGCGGTGACAGTAAA |
| AJ421962.1 | 18S_forward | TCGGCCAACAAAAGTACACA |
|  | 18S_reverse | TGTCGGTGTAACTGGCATGT |
| RPRC011390 | LpR_forward | CTCGATGAACCGAGAGCAAT |
|  | LpR_reverse | ATTCAGTTTGGCGTCTACCC |
| RPRC000551 | VgR_forward | ATTTGGACGGATTGGGGTA |
|  | VgR_reverse | TGGAGGAAAGAATGGTCCTG |
| RPRC004408 | RHBP_forward | TCCTTCACACTCTCCGCAAC |
|  | RHBP_reverse | GTACGCTTGGTACGCCACTT |
| **dsRNA** | | |
|  | T7_dsTβH_Fa | TAATACGACTCACTATAGGGAGATTGGGTATCGGCTGATTTTC |
|  | T7_dsTβH_Ra | TAATACGACTCACTATAGGGAGAGCATTGTTTCCTCGCTCTTC |
|  | T7_dsTβH_Fb | TAATACGACTCACTATAGGGAGATATCGGCTGATTTTCCATCC |
|  | T7_dsTβH_Rb | TAATACGACTCACTATAGGGAGACGGTTGCCCAAACTAGATGT |
