# Supplementary material for: Octopamine is required for successful reproduction in the classical insect model, Rhodnius prolixus
Source: PLoS One. 2024 Jul 12;19(7):e0306611. doi: 10.1371/journal.pone.0306611 (PMC11244822; doi:10.1371/journal.pone.0306611)
Supplement: S1 Raw image — (PDF) [file pone.0306611.s005.pdf]

Anti-Vg antibody

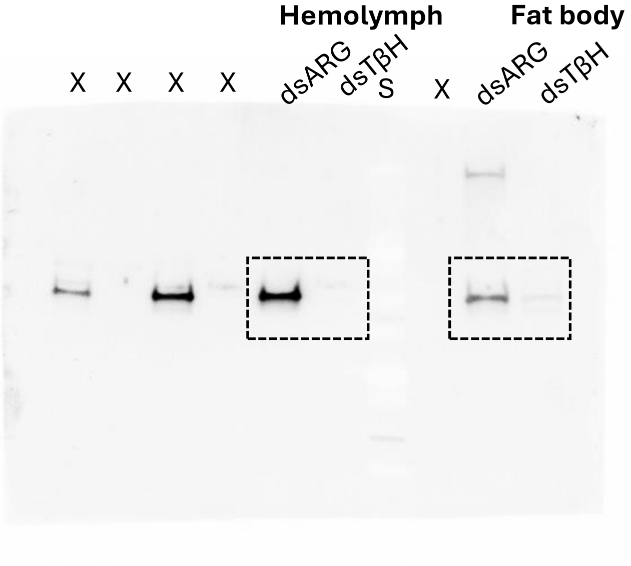

Anti-tubulin antibody

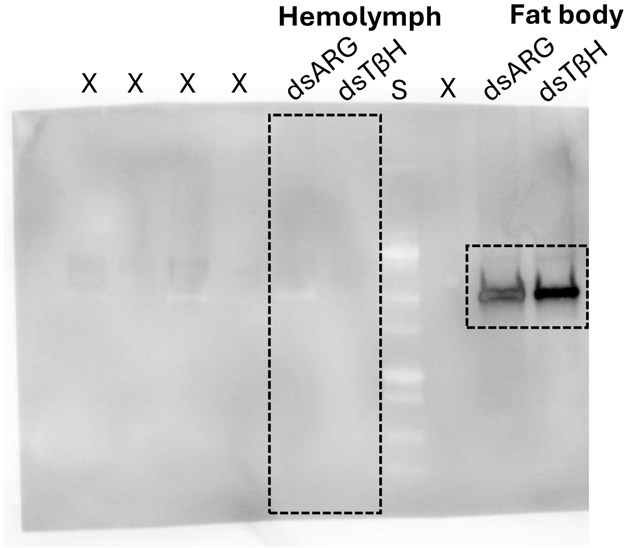

Anti-Vg antibody

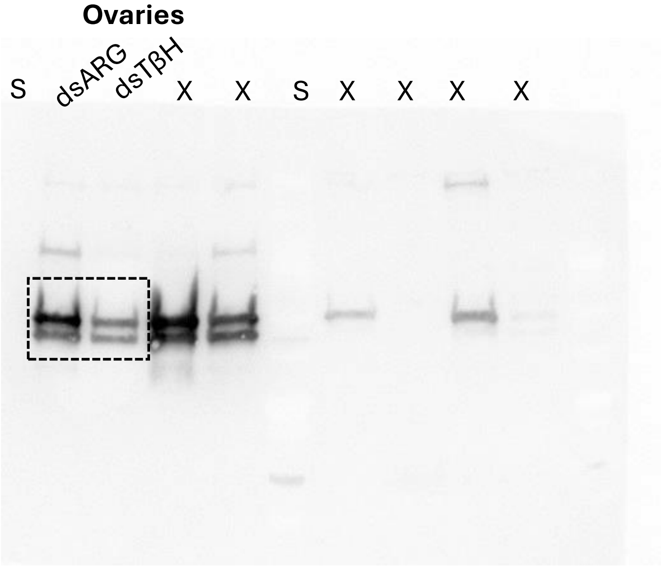

Anti-tubulin antibody

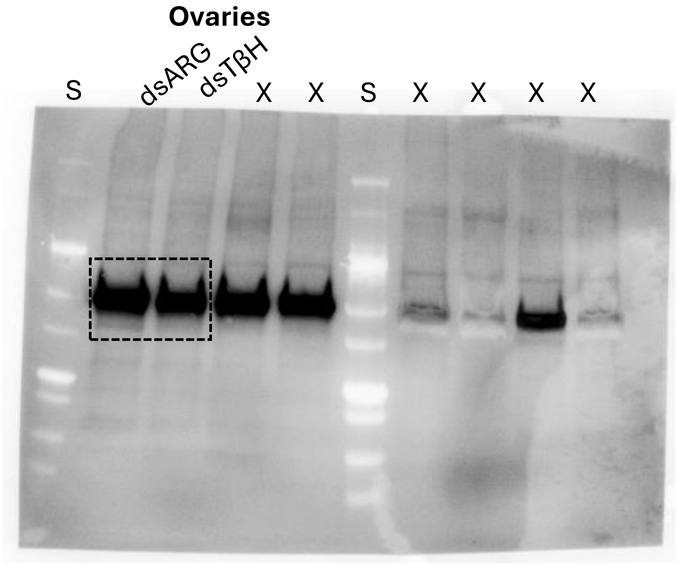

The uncropped images for western blots (5 μg/lines) shown in Fig 5 and S3 Fig. Gels were separated under reducing conditions on pre-made gels (4–20%, Mini-Protean TGX Stain-Free Precast Gels, BioRad, Mississauga, ON, Canada). Blots were probed first with the anti-Vg antibody (Boster Biological Technology (Pleasanton, CA, USA); after stripping with RestoreTM PLUS, Western blot Stripping buffer (Thermo Fisher Scientific, Mississauga, ON, Canada), the blots were then re-probed with the anti-tubulin antibody (mouse monoclonal antibody from Life Technologies, ON,

CA). Blots were visualized using enhanced chemiluminescence (Clarity Western ECL Substrate, BioRad), imaged on a ChemiDoc XRS system, and analyzed using Image Lab 5.0 (BioRad Software and System). The standards (Precision Plus Protein™ Dual Xtra Prestained Protein Standards, BioRad) were detected by fluorescence when excited at red and green wavelengths. He, hemolymph; Vg, vitellogenin; dsARG, double stranded ampicillin resistance gene; dsTßH, double stranded tyramine ß-hydroxylase; S, Protein Standards
